# MAPK-Activated Protein Kinase 2 Is Required for Mouse Meiotic Spindle Assembly and Kinetochore-Microtubule Attachment

**DOI:** 10.1371/journal.pone.0011247

**Published:** 2010-06-28

**Authors:** Ju Yuan, Bao-Zeng Xu, Shu-Tao Qi, Jing-Shan Tong, Liang Wei, Mo Li, Ying-Chun Ouyang, Yi Hou, Heide Schatten, Qing-Yuan Sun

**Affiliations:** 1 State Key Laboratory of Reproductive Biology, Institute of Zoology, Chinese Academy of Sciences, Beijing, China; 2 Department of Veterinary Pathobiology, University of Missouri-Columbia, Columbia, Missouri, United States of America; Roswell Park Cancer Institute, United States of America

## Abstract

MAPK-activated protein kinase 2 (MK2), a direct substrate of p38 MAPK, plays key roles in multiple physiological functions in mitosis. Here, we show for the first time the unique distribution pattern of MK2 in meiosis. Phospho-MK2 was localized on bipolar spindle minus ends and along the interstitial axes of homologous chromosomes extending over centromere regions and arm regions at metaphase of first meiosis (MI stage) in mouse oocytes. At metaphase of second meiosis (MII stage), p-MK2 was localized on the bipolar spindle minus ends and at the inner centromere region of sister chromatids as dots. Knockdown or inhibition of MK2 resulted in spindle defects. Spindles were surrounded by irregular nondisjunction chromosomes, which were arranged in an amphitelic or syntelic/monotelic manner, or chromosomes detached from the spindles. Kinetochore–microtubule attachments were impaired in MK2-deficient oocytes because spindle microtubules became unstable in response to cold treatment. In addition, homologous chromosome segregation and meiosis progression were inhibited in these oocytes. Our data suggest that MK2 may be essential for functional meiotic bipolar spindle formation, chromosome segregation and proper kinetochore–microtubule attachments.

## Introduction

A fundamental property of life is the ability to reproduce. Mitosis and meiosis are essential for development and utilized by organisms to pass on their genetic information. The basic factors and mechanisms governing progression through mitosis and meiosis are the same [1], [2]. However, the first meiotic division (meiosis I) is unique in that homologous chromosome segregation occurs. The second meiotic division (meiosis II) resembles mitosis in that the sister chromatids segregate.

Cell division is a multi-stage precisely orchestrated and orderly process regulated by many factors. First, the assembly of a functional spindle is critical for accurate chromosome segregation. The number and stability of microtubules nucleated from MTOCs change throughout the cell cycle, which is correlated with the assembly of the mitotic spindle [3], [4]. Spindle assembly involves coordinated activities of multiple proteins resulting in localized microtubule nucleation, dynamics, and organization, including Plk1 [5], Aurora A [6], and Astrin [7]. Second, for accurate segregation at the onset of anaphase, chromosomes need to attach, through their kinetochores, to microtubules and align at the metaphase plate [8]. The spindle assembly checkpoint (SAC) is the surveillance mechanism to ensure that anaphase onset is delayed until all chromosomes are correctly bound to microtubules [9], [10]. Third, the cohesin protein complex is essential for cohesion in both mitosis and meiosis, and cleavage of one of the subunits is sufficient for chromosome segregation at anaphase [11]. In meiosis, it is generally assumed that the normal mitotic cohesin cohort of RAD21/SCC1, SMC1α, SMC3, STAG1/SA1 and STAG2/SA2 are complemented by the meiosis-specific components REC8, SMC1β and STAG3 [12]. Meiotic sister chromatids lose cohesin from their arms until the anaphase I onset, and this is mediated solely by REC8 degradation through separase activity rather than dissociation [13], [14]. The anaphase-promoting complex/cyclosome (APC/C)[15], phosphorylation of REC8 by Polo-like kinase 1 [16] and mediated by Aurora kinase B [17], [18] are required for the cleavage-independent dissociation of cohesion from chromosomes. In mouse oocytes, SGO2 appears to be the key protector of centromeric REC8 [19], [20].

Mitogen-activated protein kinase (MAPK) signal transduction pathways are among the most widespread mechanisms of eukaryotic cell regulation that play a crucial role in many vital biological processes such as cell proliferation, cell differentiation, and cell cycle regulation. Studies in the last decade revealed that MAPK cascade also plays pivotal roles in regulating the meiotic cell cycle progression of oocytes [21], [22], specifically microtubule organization and spindle assembly during mammalian oocyte meiosis [23], [24], [25]. A subfamily of p38 MAPKs are coordinately activated in response to a wide range of extracellular stress stimuli, including cytokines and growth factors [26], [27]. The biological functions of p38 include a role in inflammatory immune responses [28] and cell cycle checkpoint controls [29], [30]. MAPK activated protein kinase 2 (MK2) is a direct substrate of p38 MAPK, and phosphorylation of MK2 by p38 MAPK results in the activation of MK2 kinase activity; in addition, MK2 determines the subcellular localization of p38 MAPK [31]. A wide variety of substrates has been described for MK2 including proteins interacting with the cytoskeleton, such as small heat shock protein Hsp25 [32]; mRNA-binding proteins, such as tristetraprolin (TTP) [33], [34]; transcription factors, such as heat shock factor 1 [35]; and regulators of the cell cycle and apoptosis, such as Cdc25B/C [36]. Moreover, MAPKAP kinase-2, a direct downstream target of p38 SAPK, is directly responsible for phosphorylating Cdc25B and C and for maintaining the G1, S, and G2/M checkpoints in response to UV-induced DNA damage [37].

In this study, we analysed the roles of MK2 in mouse oocyte meiotic maturation. We show for the first time that MK2 may regulate bipolar spindle stability, microtubule-kinetochore attachments and chromosome segregation to participate in meiotic cell cycle regulation.

## Results

### Expression and subcellular localization of p-MK2 during mouse oocyte meiotic maturation

**Figure 1:**
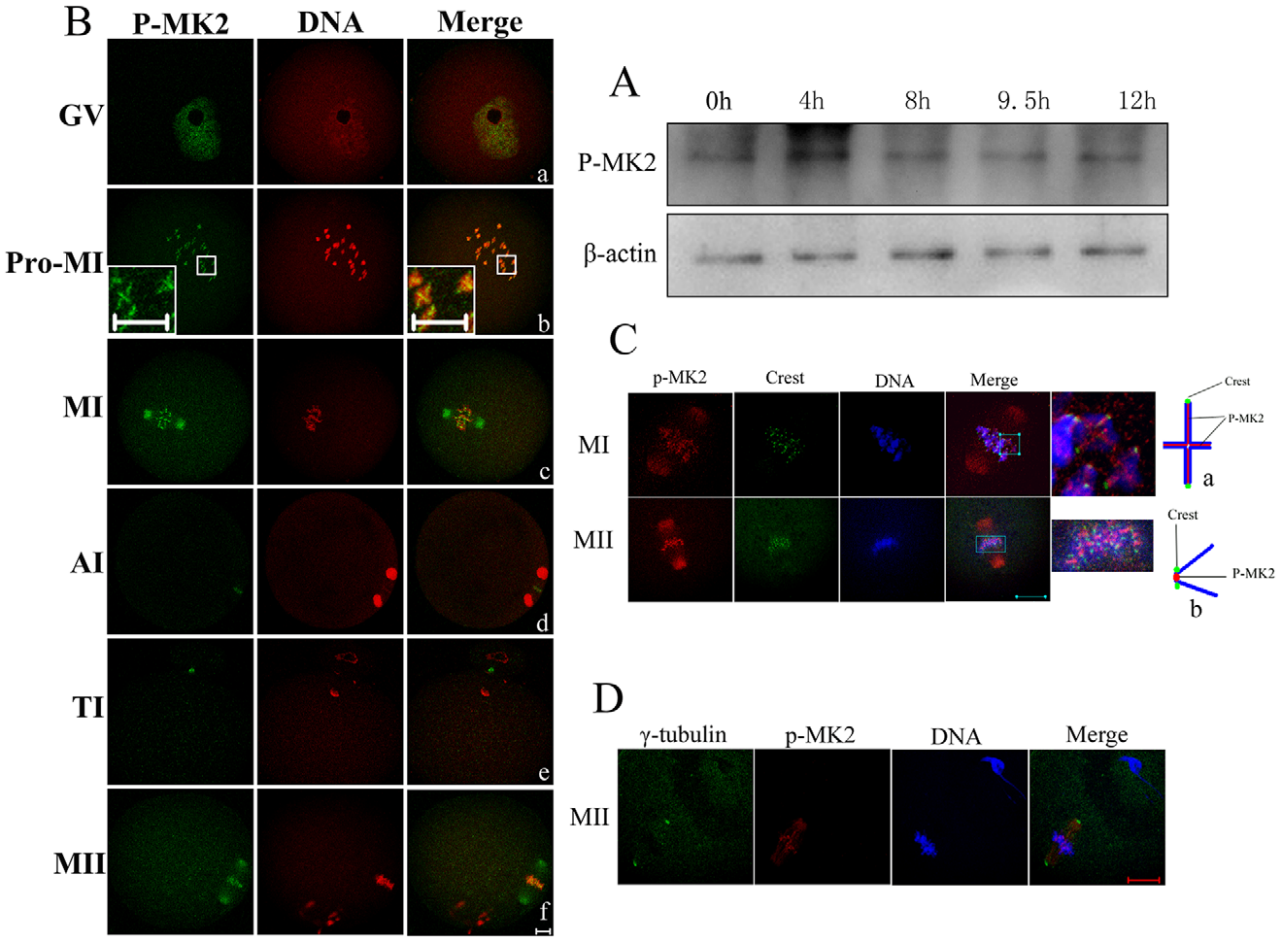
Expression and subcellular localization of p-MK2 during mouse oocyte meiotic maturation. Samples were collected after oocytes had been cultured for 0, 4, 8, 9.5 and 12 h, corresponding to GV, Pro-MI, MI, AI to TI and MII stages, respectively. Proteins from a total of 200 oocytes were loaded for each sample. (B) Oocytes at various stages were double stained with antibodies against p-MK2 (green) and with propidium iodide (PI, red). GV, oocytes at germinal vesicle stage; Pro-MI, oocytes at first prometaphase; MI, oocytes at first metaphase; AI, oocytes at anaphase; TI, oocytes at telophase; MII, oocytes at second metaphase. Bar 20 µm. (C) Metaphase I and metaphase II oocytes were fixed and double labeled with rabbit p-MK2 antibody (red) and human Crest antibody (green). Each sample was counterstained with Hoechst 33258 to visualize DNA (blue). Bar 20 µm. (a) model chart for localization of p-MK2, Crest and chromosomes at MI stage oocytes. (b) model chart for localization of p-MK2, Crest and chromosomes at MII stage oocytes. (D) Oocytes cultured for 12h (MII) were fixed and stained for γ- tubulin (green), p-MK2 (red) and DNA (blue) as visualized with Hoechst 33258 staining. Bar 20 µm.

MAPKAPK 2 (MK2) is a direct target of p38 MAPK. Multiple residues of MK2 are phosphorylated *in vivo* in response to stress. Phosphorylation at Thr222, Ser272 and Thr334 appears to be essential for the activity of MK2 [38]. Phospho-MAPKAPK-2(Thr334) antibody (p-MK2) detects endogenous levels of MK2 protein only when phosphorylated at threoine 334. To examine the expression level of p-MK2 in mouse oocytes at different stages of meiotic maturation, samples were collected after oocytes had been cultured for 0, 4, 8, 9.5, and 12 h, corresponding to germinal vesicle (GV), prometaphase I (Pro-MI), metaphase I (MI), telophase I (TI), and metaphase II (MII) stages, respectively. The immunoblotting results showed that the expression level of p-MK2 was similar at all stages (Fig. 1A).

**Figure 2:**
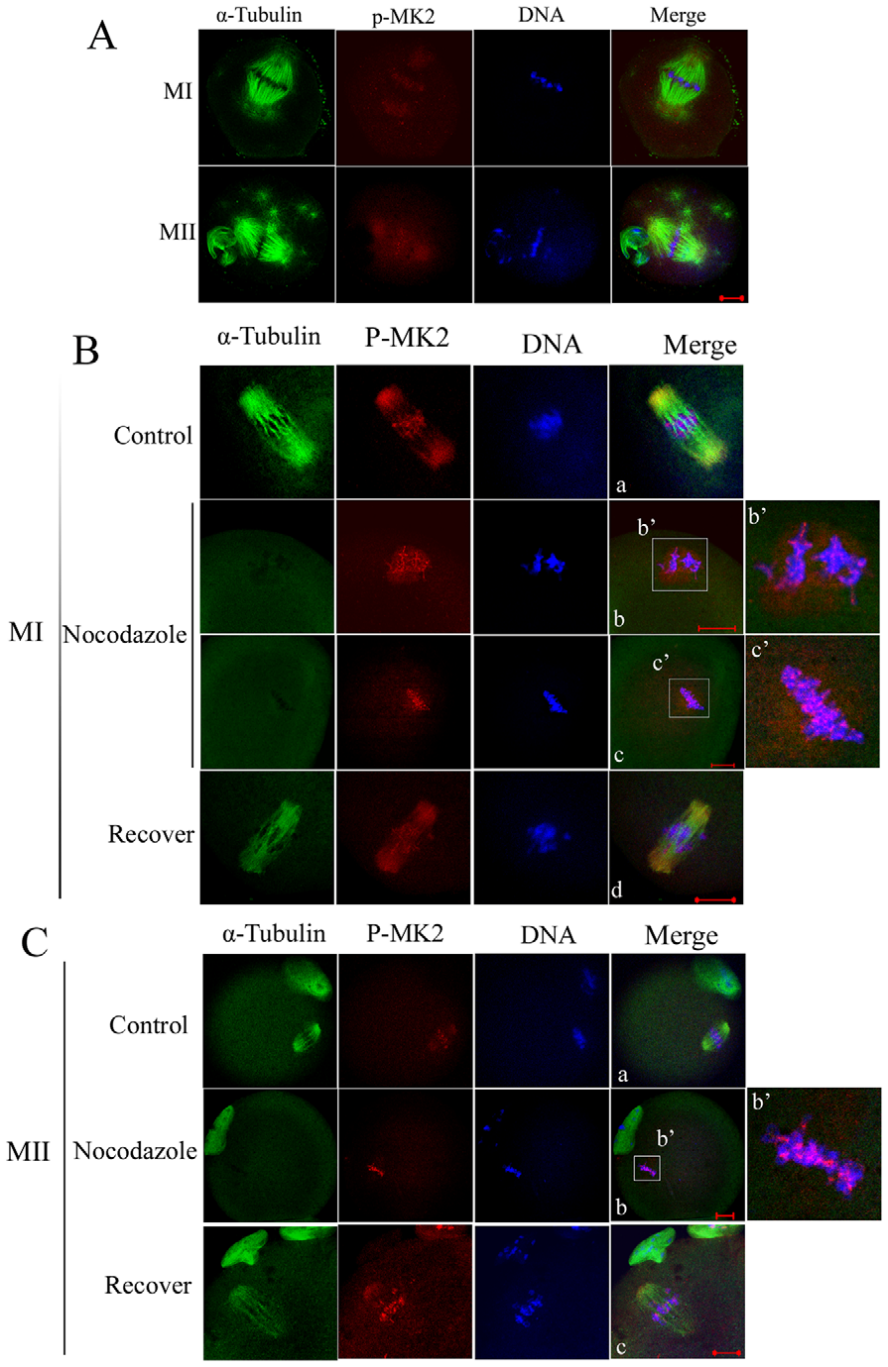
Localization of p-MK2 in mouse oocytes treated with taxol and nocodazole. (A) Oocytes cultured for 8.5 h (MI) and 12 h (MII) were incubated in M16 medium containing 10 µM taxol for 45 min and then double stained with antibodies against p-MK2 (red), α-tubulin (green), and DNA (blue). (B) Oocytes at the metaphase I stage were incubated in M16 medium containing 20 µg/ml nocodazole for 10 min (b,c) and then washed thoroughly and cultured in fresh M16 mediun for 30 min (d). Control groups were treated with DMSO (a). Then oocytes were fixed and double stained with antibodies against p-MK2 (red), α-tubulin (green), and stained for DNA (blue). Bar 20 µm. (C) Oocytes at the metaphase II stage were incubated in M16 medium containing 20 µg/ml nocodazole for 10 min (b) and then washed thoroughly and cultured in fresh M16 medium for 30 min (c). Control group was treated with DMSO(a). Then oocytes were fixed and double stained with antibodies against p-MK2 (red), α-tubulin (green), and DNA (blue). Bar 20 µm.

To investigate the subcellular localization of p-MK2, mouse oocytes were processed for immunofluorescent staining at different stages of maturation. As shown in Figure 1B, in immature mouse oocytes classified as the germinal vesicle (GV) stage, p-MK2 signals appeared as numerous dots associated with chromatin in the nucleus (Fig. 1Ba). After germinal vesicle breakdown (GVBD), in prometaphase I, chromosomes began to migrate to the equator of the spindle, and p-MK2 was localized along the interstitial axes of homologous chromosomes extending over centromere regions and arm regions, like “+” (Fig. 1Bb). When oocytes progressed to metaphase I, homologous chromosomes aligned at the equatorial plate, and p-MK2 localized at the spindle minus ends and chromosomal axes (Fig. 1Bc). At anaphase I, the homologous chromosomes were segregated, and the p-MK2 signals disappeared from chromosomes and localized to the equatorial region between the separating chromosomes (Fig. 1Bd). At telophase I, the p-MK2 signals were associated with the midbody between the oocyte and the first polar body (Fig. 1Be). At metaphase II, the p-MK2 signals again translocated to the spindle minus ends and the sister chromatids (Fig. 1Bf). For closer analysis of its localization on chromosomes (chromosome p-MK2), oocytes cultured to MI and MII stages were co-stained with p-MK2 and Crest antibodies (Fig. 1C). At the MI stage, p-MK2 localized along the interstitial axes of homologous chromosomes extending over centromere and arm regions, like “+”, and the Crest signals appeared as dots on the kinetochores (Fig. 1Ca); at the MII stage, p-MK2 localized as dots at the center of two Crest signals, that is, the inner centromere region of sister chromatids (Fig. 1Cb). Notably, when p-MK2 and γ-tubulin were co-stained at the MII stage, it was clearly observed that p-MK2 signals were not concentrated and overlapping with γ-tubulin at the centrosome position (Fig. 1D). Furthermore, when the bipolar meiotic spindles were assembled during the MI and MII stages, the p-MK2 signals were detected at the spindle minus ends, but not spread over the entire bipolar spindles, just near the minus ends of spindles (cytoplasmic p-MK2) (Fig. 2Ba,Ca); therefore, we refer to the cytoplasmic p-MK2 signals as being localized to the spindle minus ends.

In addition, p-MK2 localization at metaphse of meiosis II was similar to that of metaphase in mitosis (Fig. S1). These data imply that MAPKAP kinase 2 may be involved in bipolar spindle formation and chromosome segregation in mammalian meiosis.

### Localization of p-MK2 in mouse oocytes treated with spindle-perturbing drugs

To further clarify the correlation between p-MK2 and microtubule dynamics, spindle-perturbing drugs were employed. First, taxol was used to treat oocytes, which stabilized microtubules and reduced the tension between microtubules and kinetochores [39], [40]. When oocytes were cultured for 8.5 h and 12 h, corresponding to MI and MII stages, respectively, the microtubule fibers in taxol-treated oocytes became excessively polymerized, leading to significantly enlarged spindles and numerous asters in the cytoplasm. At MI and MII stages, p-MK2 signals were clearly detected on the chromosomes and dispersed at the two ends of stable bipolar spindles, however, p-MK2 signals were not detected in the cytoplasmic asters (Fig. 2A). These findings indicate that MK2 is required for bipolar spindle assembly but it is not a centrosome component. Moreover, the localization of p-MK2 on chromosomes was not affected by reducing the tension between microtubules and chromosomes (Fig. 2A).

When oocytes cultured to the MI or MII stage were treated with nocodazole, an inhibitor of tubulin polymerization, the microtubules were completely disassembled. Compared to control oocytes (Fig. 2Ba), the p-MK2 signals disappeared from the spindle and dispersed around the chromosomes at the MI stage (Fig. 2Bb,c). At the MII stage, the cytoplasmic p-MK2 signals also disappeared from the spindle and no visible signals were detected around the chromosomes (Fig. 2Cb), compared to control oocytes (Fig. 2Ca). Meanwhile, the chromosome p-MK2 signals were still detected on the chromosomes, when microtubule and chromosome attachments were completely destroyed (Fig. 2Bb,c, Cb). In addition, when the above-described nocodazole-treated oocytes were cultured in fresh medium for 30 min, the cytoplasmic p-MK2 relocated to the spindle minus ends when the bipolar spindle was re-assembled (Fig. 2Bd,Cc). We conclude that MK2 is involved in maintaining bipolar microtubule organization while microtubule disassembly does not affect MK2 function on chromosomes.

### Depletion of MK2 by morpholino injection leads to spindle defects, homologous chromosome nondisjunction and meiosis progression arrest

**Figure 3:**
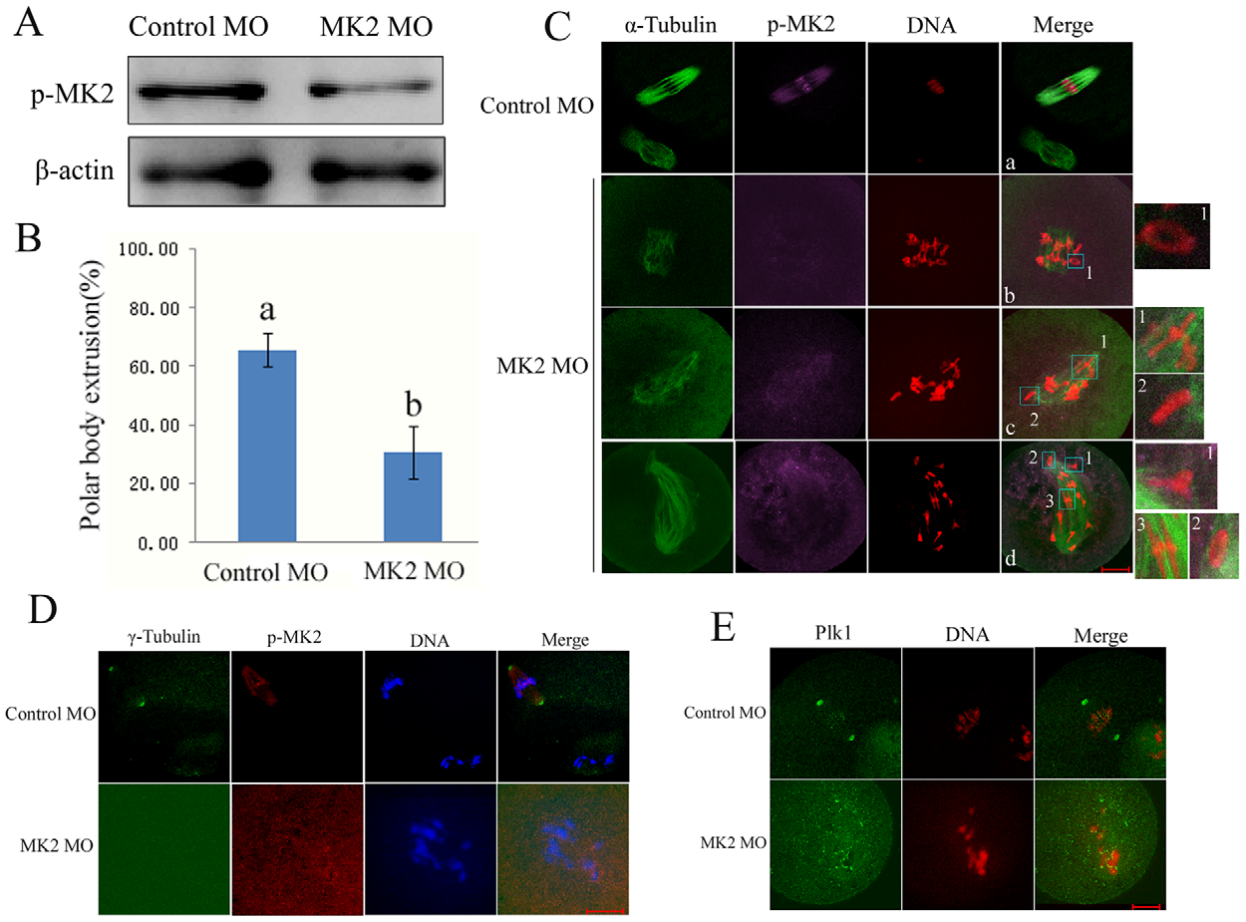
Depletion of MK2 by morpholino injection induced homologous chromosome misalignment and bipolar spindle assembly defects. (A) Samples from control and MK2 MO injection groups were collected to test the efficiency of MK2 depletion. A total of 200 oocytes were injected with control or MK2 morpholino and cultured for 6 h after arrest at the GV stage for 24 h in M2-containing 2.5 µM milrinone. (B) Percentage of polar body extrusion in the MK2 morpholino injected group (n = 196) and control morpholino injected group (n = 137). Data are presented as mean ± SE. Different superscripts indicate statistical difference (p<0.05). (C) Immunostaining of oocytes injected with control and MK2 morpholino. After injection, oocytes were incubated for 12 h after arrest at the GV stage for 24 h, followed by immunostaining with α-tubulin antibody (green), p-MK2 (purple) and PI labeling for DNA (red). Bar 20 µm. (D) The above oocytes were immunostained with γ- tubulin (green), p-MK2 (red) and labeled for DNA (blue). Bar 20 µm. (E) The above oocytes were immunostained with Plk1 (green) and labeled with PI (red). Bar 20 µm.

To elucidate the physiological function of MK2 in oocyte maturation, we employed a morpholino-based gene silencing approach to deplete MK2 in oocytes. Negative control or MK2 morpholino was injected into GV stage oocytes and maintained in 2.5 µM milrinone for 24 h, and then transferred to fresh M2 medium for 6 h. As presented in Fig. 3A, compared to the control morpholino (MO) group, the MK2 expression in MK2 MO-injected oocytes was significantly reduced, revealing successful down-regulation of MK2. When the MK2 MO-injected oocytes were cultured for 12 h after release from the inhibitory environment, there was a marked reduction in the polar body extrusion (PBE) rate. Only 30% of MK2-depleted oocytes underwent PBE, which was significantly lower than that of the control group (65%) (p<0.05, Fig. 3B). Furthermore, immunofluorescent staining showed that most control MO-injected oocytes which extruded the PB (85.6%, n = 70) displayed normal-looking spindles and sister chromatids arranged at the middle plate (Fig. 3Ca), however, most (90.4%, n = 89) MK2 MO-injected oocytes displayed abnormal spindles and homologous chromosome congression defects, such as irregularly scattered chromosomes surrounding shrinking or collapsed spindles (Fig. 3Cb), or normal-looking spindles with scattered misaligned chromosomes (Fig. 3Cc) or scattered chromosomes along the elongated spindle (Fig. 3Cd). Moreover, in the control MO-injected group, MK2 signals were observed at spindle ends and centromeres of sister chromatids, whereas in the MK2 MO-injected group, most of the MK2 signals could not be detected at the homologous chromosomes and microtubules. Notably, the morphology of the homologous chromosomes in MK2-deficient oocytes was different from the control (Fig. 3Cb1,c1–2,d1–3). These results revealed that down-regulation of MK2 results in spindle assembly defects, homologous chromosome nondisjunction and misalignment.

We examined the localization of the centrosome protein γ- tubulin and the centrosome-associated protein kinase Plk1 in MK2 MO-injected oocytes. As presented in Fig. 3D, the centrosome protein γ- tubulin disappeared from the cytoplasm unlike in control oocytes in which γ- tubulin was localized at the spindle poles, and the level of p-MK2 was significantly reduced in MK2 MO-injected oocytes. Fig. 3E shows that Plk1 was only detected at the kinetochores compared to control oocytes in which Plk1 was localized at spindle poles and kinetochores. These results suggest that the MK2-deficient oocytes contain a collapsed spindle without spindle poles.

From the data above, we conclude that depletion of MK2 by morpholino injection caused meiosis progression arrest with damaged spindles and nondisjuncted chromosomes. In addition, these results were further proved by RNAi depletion of MK2 (Fig. S2).

### MK2 inhibitor treatment results in spindle defects and homologous chromosome nondisjunction similar to depletion of MK2 by morpholino injection

**Figure 4:**
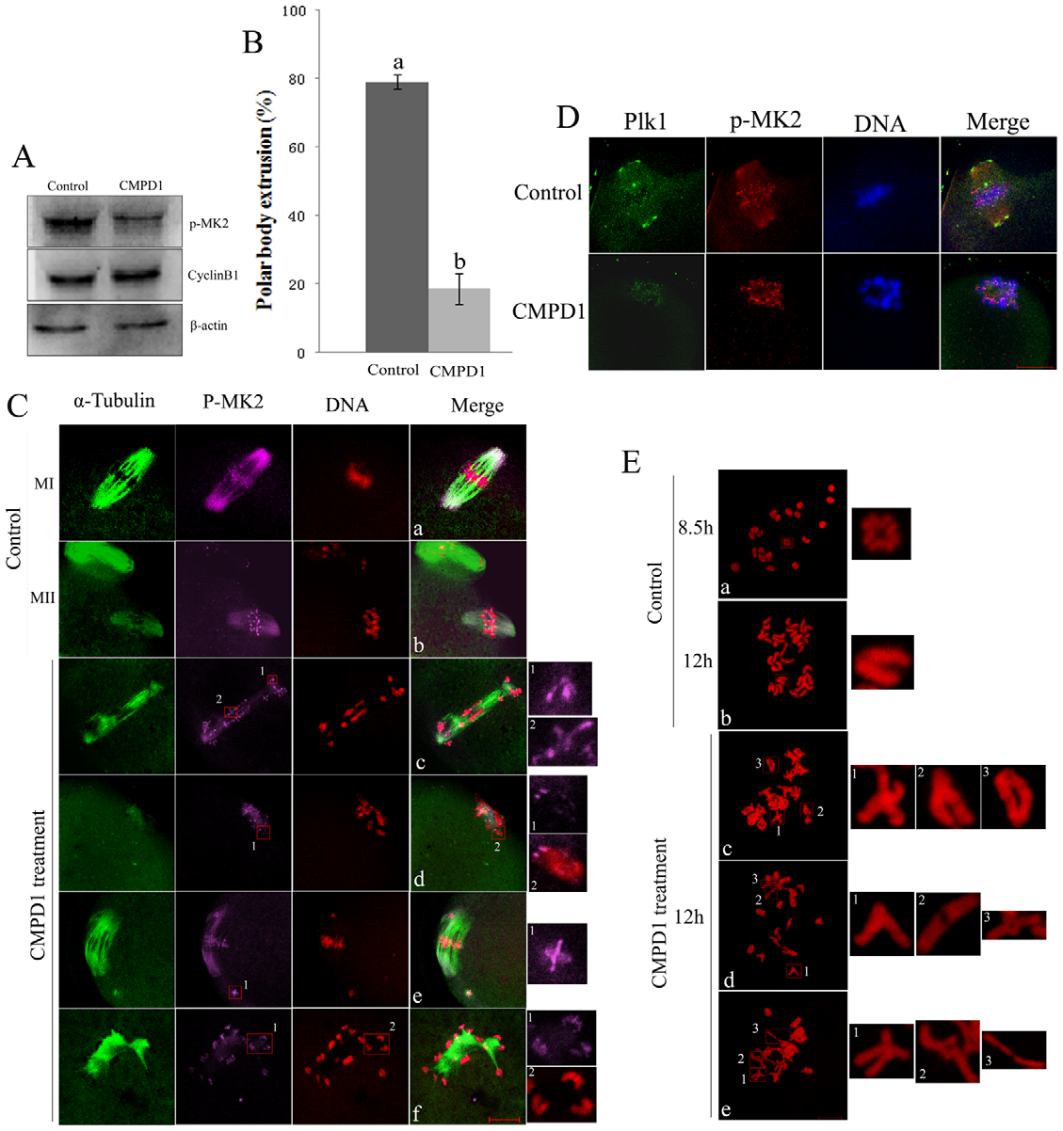
MK2 inhibitor CMPD1 treatment impairs spindle organization and chromosome alignment in mouse oocytes. (A) Samples from control and CMPD1 groups were collected to test the efficiency of the MK2 inhibitor. CMPD1, 200 oocytes were cultured in M16 medium with 30 µM CMPD1 for 10 h; Control, 200 oocytes were cultured in M16 medium with DMSO for 10h. (B) The rate of oocytes with first polar body in the control group (n = 267) and CMPD1-treated group (n = 318). Data are presented as mean percentage (mean ± SEM) of at least three independent experiments. Different superscripts indicate statistical difference (p<0.05). (C) Spindle morphologies and chromosome alignment in oocytes cultured with DMSO or MK2 inhibitor CMPD1. GV oocytes were cultured in M16 medium with DMSO or with 30 µM CMPD1 for 12 h and then stained for p-MK2 (purple), α-tubulin (green) and DNA (red). Bar 20 µm. (D) GV oocytes were cultured in M16 medium with DMSO or with 30 µM CMPD1 for 8.5 h and then stained for p-MK2 (red), Plk1 (green) and DNA (blue). Bar 20 µm. (E) Chromosome spreading was performed in oocytes that had been cultured for 8.5 h or 12 h of DMSO treatment (MI) (MII) or for 12 h of CMPD1 treatment. Representative images of each sample are shown. Bar 10 µm.

CMPD1, an MK2α specific p38α inhibitor, is noncompetitive with ATP and specifically prevents the phosphorylation and activation of MK2α by p38α [41]. To further investigate the function of MK2 during oocyte maturation, the inhibitor CMPD1 was used to prevent phosphorylation of MK2. GV oocytes were incubated in M16 medium containing 30 µM CMPD1 for 11 h, and then washed thoroughly for Western blot analysis. As shown in Figure 4A, the p-MK2 protein expression level was strikingly reduced compared to the control, which showed that CMPD1 had successfully prevented phosphorylation of MK2. In addition, the cyclin B1 level in the CMPD1-treated oocyte group was not reduced compared to control oocytes, which showed that preventing the phosphorylation level of MK2 had impaired meiosis progression (metaphase-to-anaphase transition). Most oocytes of the CMPD1-treated group did not extrude the first polar body even if they were cultured for 12 hours, indicating that inhibition of MK2 had greatly impaired meiosis progression (Fig. 4B). Compared to control MI and MII oocytes which displayed normal looking spindles and regular chromosomes (Fig. 4Ca,b), almost all (86.4%, n = 217) of the CMPD1 treated oocytes cultured for 12 h displayed abnormal spindles with dispersed homologous chromosomes (Fig. 4C). Some oocytes showed lagging chromosomes or irregularly scattered chromosomes surround shrinking or collapsed spindles (Fig. 4Cc); some showed dished chromosomes surrounding the shrinking spindles (Fig. 4Cd); some showed irregularly scattered chromosomes with normal spindles (Fig. 4Ce); and some showed monopolar spindles surrounded by homologous chromosomes (Fig. 4Cf). The chromosome p-MK2 staining was still observed along the centromere and arm regions of homologous chromosomes, and the cytoplasmic p-MK2 had nearly disappeared from the abnormal spindles (Fig. 4Cc-f). However, the morphology of homologous chromosomes was abnormal and different from each other as revealed by staining with p-MK2 and DNA labeling (Fig. 4Cc1-2,d1-2,e1,f1-2). We next examined the localization of centrosome-associated protein kinase Plk1 in CMPD1-treated oocytes. As presented in Fig. 4D, the centrosome-associated protein Plk1 was only detected at the kinetochores compared to control oocytes in which Plk1 was localized at spindle poles and kinetochores, indicating that the CMPD1 treatment caused collapse of spindles with no apparent spindle poles.

Notably, the MK2-deficient oocytes in both the inhibitor-treated group and the morpholino injection group showed various kinds of chromosome morphologies. To examine details in chromosome morphology, CMPD1-treated oocytes were used for chromosome spreading. In meiosis, the chromosomes of oocytes cultured for 5–8.5 hours displayed 20 bivalents, like“+” (Fig. 4Ea); when oocytes were cultured for 12 h, the bivalents separated and became 20 univalents (Fig. 4Eb). Notably, the CMPD1-treated oocytes cultured for 12 h showed abnormal 20 bivalents (Fig. 4Ec-e), and the morphology of chromosomes in most (75.4%, n = 57) oocytes was abnormal compared to the controls. The homologous chromosomes may not have been able to become segregated, perhaps due to errors in microtubule-kinetochore attachments and tension. However, meiosis progression of CMPD1-treated oocytes can be recovered by culture for 5 h in fresh M2 medium after CMPD1 treatment for 10 h. The recovered oocytes successfully extruded the first polar body (Fig. S3A), however, the recovered oocytes (81.6%, n = 98) displayed abnormal spindles with dispersed chromosomes compared to control MII oocytes (Fig. S3B).

These results further show that MK2 is involved in microtubule dynamics, chromosome segregation and correct microtubule-kinetochore attachment.

### CMPD1 treatment leads to metaphase II spindle assembly defects, misaligned chromosomes and aneuploidy

**Figure 5:**
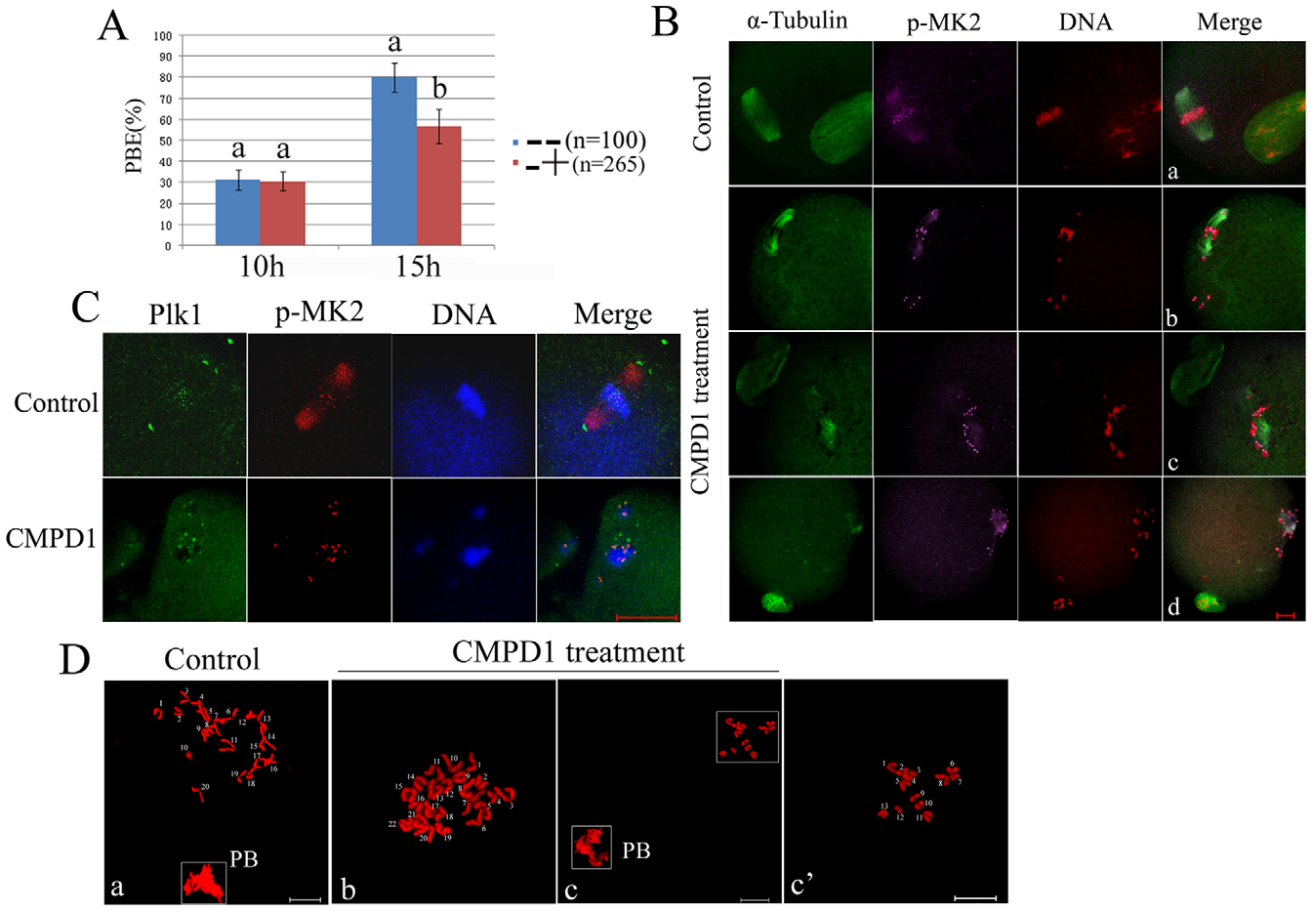
CMPD1 treatment leads to metaphase II spindle assembly defects, misaligned chromosomes and aneuploidy. (A) The rate of oocytes with first polar body in the control group and CMPD1-treated group. Control group (−−): oocytes cultured with DMSO for 15 h (n = 100); CMPD1-treated group (−+): oocytes cultured with DMSO for 10 h, and then with 30 µM CMPD1 for 5 h (n = 265). Data are presented as mean percentage (mean ± SEM) of at least three independent experiments. Different superscripts indicate statistical difference (p<0.05). (B) Spindle morphologies and chromosome alignment in the oocytes first cultured with DMSO for 10 h, and then with MK2 inhibitor CMPD1 for 5 h; then stained for p-MK2 (purple), α-tubulin (green) and DNA (red). Control group oocytes were cultured with DMSO for 15 h. Scale bar, 20 µm. (C) GV oocytes were treated as in (B), and then stained for p-MK2 (red), Plk1 (green) and DNA (blue). Bar 20 µm. (D) Chromosome spreading was performed in oocytes that had been treated as (B). Representative images for each sample are shown. Bar 10 µm.

As shown above, MK2 morpholino injected oocytes or MK2 inhibitor CMPD1-treated oocytes showed meiosis progression arrest at the metaphase I stage. In order to explore the MK2 function in second meiotic division, MK2 inhibition was used to treat oocytes cultured for 10 h when most oocytes had moved past the metaphase I stage. When the CMPD1-treated oocytes were further cultured for 5 h, the percentage of the first polar body extrusion was lower compared to that of the control group treated with DMSO, but higher than that of oocytes treated with CMPD1 at the GV stage (Fig. 5A, Fig. S2A). The staining showed that most CMPD1-treated oocytes with first polar body (78.1%, n = 128) displayed abnormal spindles and misaligned chromosomes compared to control oocytes (Fig. 5B), while some oocytes displayed normal looking spindles with scattered chromosomes (Fig. 5Bb), and some oocytes showed lagging chromosomes or irregularly scattered chromosomes surrounding shrinking or collapsed spindles (Fig. 5Bc,d). In addition, p-MK2 staining was detected at the centromeres of sister chromatids as dots and on the collapsed spindles (Fig. 5Bb-d). We further examined the localization of the centrosome-associated protein kinase Plk1 in CMPD1-treated oocytes. As presented in Fig. 5C, the centrosome-associated protein kinase Plk1 was only detected at the kinetochores compared to control oocytes in which Plk1 was localized to both spindle poles and kinetochores.

To examine details of sister chromatid segregation of CMPD1-treated oocytes at first meiosis, we employed chromosome spreading. Control oocytes cultured for 15 h contained 20 sister chromatids (84%, n = 50) (Fig. 5Da), however, most of the CMPD1-treated oocytes with the first polar body (65.2%, n = 63) showed incorrect numbers of sister chromatids with more or less than 20 (Fig. 5Db,c).

Taken together, these results indicate that at the second meiotic division, CMPD1 treated oocytes displayed defective spindle assembly. The polar body extrusion was triggered despite the presence of unattached or improperly attached chromosomes, resulting in missing or extra chromosomes (aneuploidy) in the daughter cells.

### MK2 depletion impairs correct microtubule-kinetochore attachment

**Figure 6:**
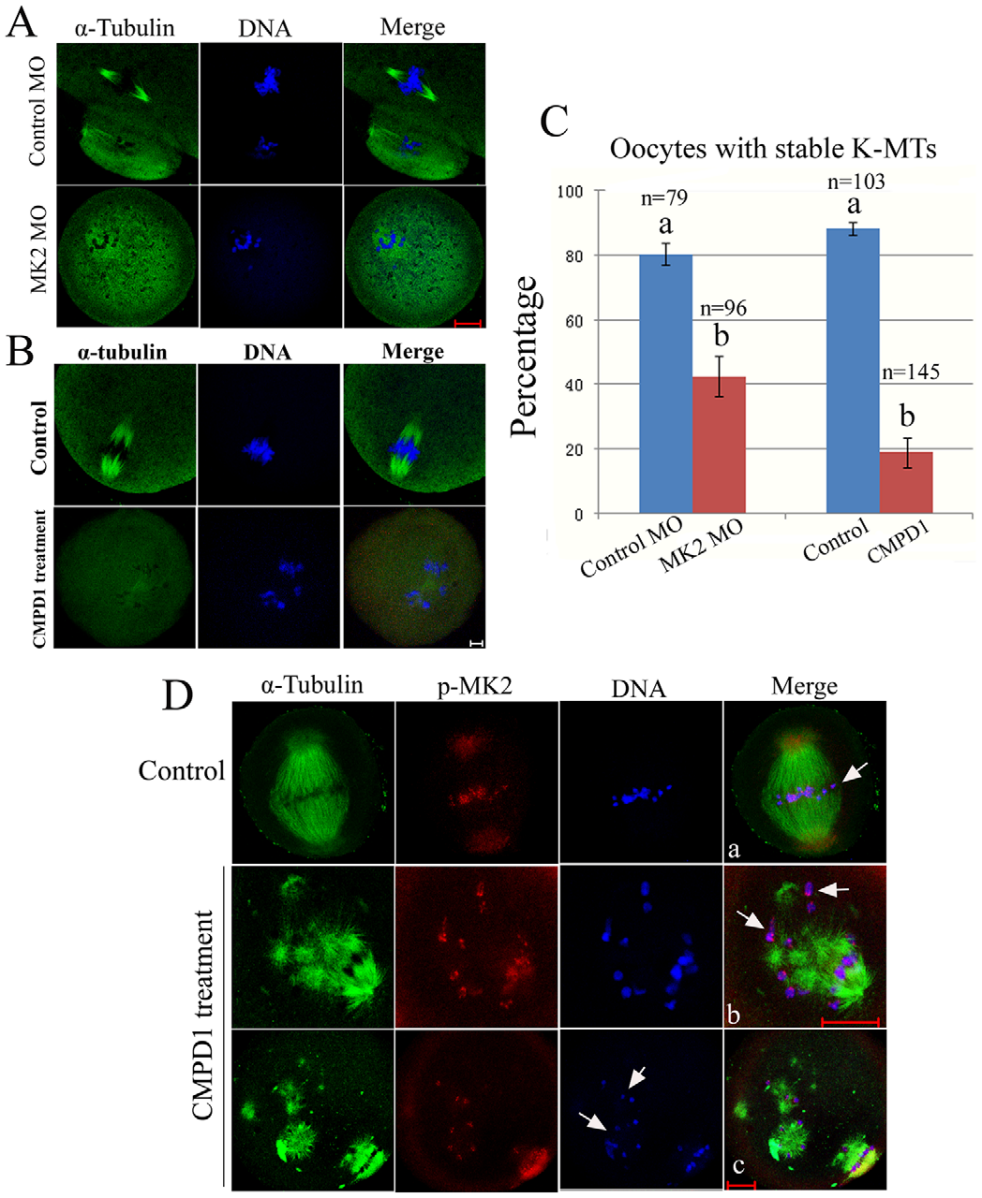
MK2 depletion interferes with correct microtubule–kinetochore attachment. (A) After culture for 12 h in M16 medium at 37°C, oocytes injected with control or MK2 morpholino were quickly transferred to ice-cold medium at 4°C for 20 min. These oocytes were fixed and stained for α-tubulin (green) and DNA (blue). Scale bar, 20 µm. (B) After 8.5 h of culture in M16 medium with DMSO or CMPD1 at 37°C, oocytes were quickly transferred to ice-cold medium at 4°C for 20 min. These oocytes were fixed and stained for α-tubulin (green) and DNA (blue). Scale bar, 10 µm. (C) Percentages of oocytes with stable K-MTs after cold treatment as in (A,B). (D) Oocytes cultured in medium with DMSO or CMPD1 for 8.5 h were incubated in M16 medium containing 10 µM taxol for 45 min and then double stained with antibodies against p-MK2 (red), α-tubulin (green), and DNA label (blue). Bar 20 µm. White arrowhead indicates homologous chromosome attachment to microtubules.

As shown above, MK2 inhibition or depletion resulted in collapsed spindles, and chromosomes appeared to be excluded from the spindle structure or arranged in a dome-like configuration surrounding the spindle (Fig. 3C, Fig. 4C). These phenotypes suggest that MK2-depleted oocytes are unable to undergo proper chromosome alignment because of defects in spindles and/or kinetochore–microtubule attachments [42]. In order to confirm this hypothesis, we tested the stability of the spindle microtubules in response to cold treatment [43]. Although cold treatment moderately affected spindle morphology in control cells, the overall structure of the kinetochore attached microtubules remained robust in control cells (Fig. 6A,B). In contrast, cold treatment resulted in highly unstable and much disintegrated microtubules in MK2 MO-injected or CMPD1-treated oocytes (Fig. 6A,B,C). This result suggests that spindle microtubules are not properly attached to kinetochores in MK2-deficient oocytes.

To further confirm this speculation, we performed an assay designed to quantify microtubule–kinetochore interactions [44]. For this assay, GV oocytes were cultured in M16 medium with DMSO or CMPD1 for 8.5 h and subjected to taxol for 45 min, which causes the collapse of the bipolar spindle into a monopolar configuration. The microtubule–kinetochore interactions could be scored easily in this structure. We found that in control MI oocytes, most of the chromosomes aligned at the equatorial plate displayed both kinetochores attached to microtubules in an amphitelic configuration (Fig. 6Da). However, in the MK2-deficient oocytes, bundles of abnormal spindle microtubules were excessively polymerized, and it was clearly observed that some chromosomes localized between asters and the attachments were amphitelic; some chromosomes localized to the periphery of the aster and the attachments were syntelic/monotelic; and some chromosomes were scattered or detached from the spindle (Fig. 6Db,c). In control oocytes (n  = 112), most chromosomes (95%) exhibited a clear amphitelic configuration, whereas in MK2-depleted oocytes (n = 217), only few chromosomes (11%) were localized between the periphery of the two asters with a clear amphitelic configuration.

The collapsed and cold sensitive spindles with the scattered chromosome configurations in MK2-deficient oocytes suggests that kinetochores were not properly captured by microtubules. To determine whether the lack of microtubule–kinetochore attachment is due to defective kinetochore structure, we next examined the localization of kinetochore-associated protein kinase Plk1 to assess the integrity of the kinetochore structure in MK2-deficient oocytes. We found that Plk1 was localized properly at the kinetochores in MK2-deficient oocytes (Fig. 3D, Fig. 4D, Fig. 5C). Taken together, these results indicate that knockdown of MK2 impairs correct microtubule-kinetochore attachments but does not affect kinetochore integrity.

## Discussion

The formation of a stable bipolar meiotic spindle is crucial for chromosome disjunction in which chromosome attachment, alignment and pulling toward spindle poles are critical steps for accurate segregation. Although chromatin interaction with microtubules appears to influence microtubule dynamics, a bipolar spindle can still form in the absence of chromatin, as is the case in enucleated mouse oocytes [45], [46]. Our studies for the first time showed that phosphorylated MAPKAPK 2 (p-MK2) is localized to bipolar spindle minus ends and chromosomes during metaphase I and metaphase II in meiosis. In addition, in the Pro-MI oocytes, in which the connection between microtubules and chromosomes had not yet been completely established and the bipolar spindle had not yet formed, p-MK2 was only localized along the interstitial axes of homologous chromosomes. Furthermore, at anaphase I, p-MK2 was localized in the region of the equatorial plate between the separating chromosomes. MK2-deficient oocytes after inhibitor treatment and morpholino injection both resulted in collapsed spindles with no centrosomes apparent at the poles. These data suggest that MK2 most likely contributes to meiotic bipolar spindle assembly, similar to mitosis where MK2 functions in bipolar spindle formation shown by MK2 depletion which induced mitotic defects in bipolar spindle formation and subsequent activation of the spindle checkpoint [47]. However, in mitosis, p-MK2 localization to spindle poles during prophase and metaphase is different from our findings showing p-MK2 localization to both microtubule minus ends and chromosomes, perhaps because our p-MK2 antibody may have been more suitable for immunofluorescence.

To further clarify the correlation between p-MK2 and microtubule dynamics, we destructed bipolar spindle structures, and found that all cytoplasmic p-MK2 emerged around the chromosomes at the MI stage, but the cytoplasmic p-MK2 signals disappeared completely at the MII stage. We speculate that p-MK2 accumulated at the minus ends of microtubules and was retained at the kinetochores of homologous chromosomes and sister chromatids. Another explanation might be that p-MK2 may have different roles in first meiotic bipolar spindle and second meiotic bipolar spindle formation. There are some proteins regulating meiosis I spindle formation differently from meiosis II spindle formation, such as ATRX [48] and Ran [49]. However, we find that inhibition of MK2 at first meiotic and second meiotic division both results in collapsed spindles and unaligned chromosomes. The third explanation might be that different mechanisms are employed for meiosis I and II spindle formation [49]. In mouse oocytes, the first meiotic M phase is very long, lasting from 6 to 11 h, depending on the genetic background [50], [51]. However, the MII spindle assembles rapidly, and 1 h after first polar body emission in mouse oocytes a stable bipolar spindle has already formed.

We further observed that p-MK2 was localized to the spindle minus ends of bipolar microtubule fibers and not to cytoplasmic asters after taxol treatment. This unexpected localization of cytoplasmic MK2 suggests that it serves functions in the establishment or stabilization of the bipolar spindle, but not in microtubule nucleation or organization, which is different from functions of other microtubule nucleation-related protein kinases such as MEK and Polo-like kinase 1 [5], [52].

To our knowledge, we are the first to show that p-MK2 signals are localized along the interstitial axes of homologous chromosomes in meiosis extending over centromere and arm regions at the proMI and MI stages. Moreover, at the MII stage, p-MK2 signals were detected as centromeric dots of sister chromatids. Clearly, similar to the REC8 signals in meiosis which distributes along the entire axis of chromosomes during meiosis I, p-MK2 localization on the cohesion domains of meiotic homologous chromosomes and sister chromatids, disappears from chromosome arms at the onset of anaphase I, but persists at centromeres until the onset of anaphase II[53]. However, at the AI or TI stage, p-MK2 was no longer associated with the centromeric domains of meiotic chromosomes; therefore, we suggest that MK2 is not part of the cohesin complex and is not directly responsible for holding sister chromatids together, but it may be essential for cohesin degradation or activation, which needs further investigation.

The cohesin family of proteins play a well-established role in maintaining sister chromatid cohesion in mitosis and meiosis in a number of species [12], [54]. In contrast, meiotic sister chromatids do not lose cohesin from their arms until the anaphase I onset, and this is mediated solely by REC8 degradation through separase activity rather than dissociation [13], [14]. Recently, several proteins were reported to be involved in the regulation of REC8 degradation; for example, Aurora B kinase is localized to the chromosomal arms distal to chiasmata and leads to phosphorylation and localized destruction of the meiotic cohesin REC8 [17], [55]. Moreover, cohesin phosphorylation depends on Polo-like kinase (Plk1) and reduces the ability of cohesin to bind to chromatin; therefore, Polo-like kinase is required for the cleavage-independent dissociation of cohesin from chromosomes [16], [56]. Furthermore, MK2 has been proved to directly phosphorylate Ser326 of Plk1 [47]. We found that knock-down of MK2 by inhibitor treatment and morpholino injection both lead to homologous chromosome segregation failure and meiosis progression arrest. However, inhibition release of phosphorylated MK2 could break through the metaphase I arrest and allow extrusion of the polar body. The explanation is that MK2-deficiency reduces the level of Plk1 phosphorylation which may downregulate cohesin phosphorylation and prevent homologous chromosome separation. Because of the lack of phospho-Plk1 and REC8 antibodies, this assumption could not be confirmed further in our study.

Kinetochore microtubules (kMTs) are spindle microtubule whose minus ends are typically anchored at the spindle poles and whose plus ends terminate end-on at the kinetochore. Chromosome movement depends on the assembly dynamics of kMT plus ends within their kinetochore attachment sites [57]. Normally, kMTs are stable to cold- or calcium-induced depolymerization because of their kinetochore plus-end attachments; in contrast, most spindle MTs with free plus-ends are not stable before anaphase [43], [58]. The phenotypes of collapsed spindles with chromosomes excluded from the spindle structure or arranged in a dome-like configuration surrounding the spindle in MK2-depleted oocytes strongly suggest that down-regulation of MK2 results in failure of proper chromosome alignment because of defects in spindles and/or kinetochore–microtubule attachment [42]. Moreover, cold treatment resulted in a highly unstable and much disintegrated spindle in MK2-deficient oocytes. In taxol treated MK2-deficient oocytes, it is clearly seen that chromosome attachment is either amphitelic, syntelic/monotelic, or that chromosomes are detached from spindle asters. These results further suggest that the spindle microtubules are not properly attached to kinetochores in MK2-deficient cells.

In order for homologous chromosomes to align properly at the metaphase plate and segregate equally to daughter cells through bipolar spindle dynamics, correct microtubule–kinetochore interactions must be established to direct sister kinetochores to opposite poles (amphitelic kinetochore orientation). However, we found that various incorrect kinetochore–microtubule attachments occur in MK2 deficient oocytes. The kinetochores were either not bound to microtubules (detached), or one kinetochore was bound to microtubules while the other one was unbound (monotelic kinetochore orientation), or both kinetochores were bound to microtubules from the same pole (syntelic kinetochore orientation), and one kinetochore was bound to microtubules from both spindle poles (merotelic kinetochore orientation) (Fig. S4). If errors are not corrected before the onset of anaphase, meiosis progression is arrested. Therefore, additional regulation is needed to eliminate improper microtubule–kinetochore associations to allow for correct cell division [59]. As previously reported, the spindle assembly checkpoint Bub3 [60], [61], BubR1 [62], [63] and Bub1 [60], [64] localized to the kinetochores and is required for the establishment of efficient K-MT attachments. Moreover, histone deacetylase 3 localized to the mitotic spindle and is required for kinetochore-microtubule attachment [42]. Importantly, numerous studies revealed that Aurora B is concentrated at the centromere in prometaphase and metaphase [65] and interaction with mitotic centromere-associated kinesin (MCAK) is important for proper chromosome biorientation and for correcting or preventing improper microtubule-kinetochore attachments in mammalian cells [66], [67], [68], [69]. Our study supports the idea that MK2 may play a critical role in the establishment of correct kinetochore-microtubule attachments by phosphorylating some unknown key substrates.

In conclusion, in this study we report for the first time that MK2 localizes at the meiotic bipolar spindle microtubule minus ends and cohesion of chromosomes, and we provide evidence that MK2 is required for bipolar spindle formation, homologous chromosome segregation and proper kinetochore–microtubule attachment.

## Materials and Methods

All chemicals and media were purchased from Sigma Chemical Company (St. Louis, MO) except for those specifically mentioned.

### Ethics Statement

Animal care and use were conducted in accordance with the Animal Research Committee guidelines of the Institute of Zoology, Chinese Academy of Sciences.

### Mouse oocyte collection and culture

Immature oocytes were collected from ovaries of 6-week-old Kunming White mice in M2 medium. Only those immature oocytes displaying a germinal vesicle (GV) were cultured further in M16 medium under liquid paraffin oil at 37°C in an atmosphere of 5% CO_2_ in air. At different times of culture, oocytes were collected for immunostaining, drug treatment, or microinjection.

### Taxol and nocodazole treatment of oocytes

Oocytes were treated at various stages with taxol or nocodazole. For taxol treatment, 5mM taxol in DMSO stock was diluted in M16 medium to give a final concentration of 10 µM and oocytes were incubated for 45 min; for nocodazole treatment, 10 mg/ml nocodazole in DMSO stock was diluted in M16 medium to give a final concentration of 20 µg/ml and oocytes were incubated for 10 min. After treatment, oocytes were washed thoroughly and used for immunofluorescence. Control oocytes were treated with the same concentration of DMSO in the medium before examination.

### CMPD1 treatment and Cold treatment

For MK2 inhibitor CMPD1 (Calbiochem) treatment, 30 mM CMPD1 in DMSO stock was diluted in M16 medium to give a final concentration of 30 µM. The oocytes were incubated in M16 medium containing 30 µM CMPD1 for different times, and then washed thoroughly for subsequent experiments. Control oocytes were treated with the same concentration of DMSO in the medium before examination.

For cold treatment, oocytes at the appropriate stages were transferred to M16 medium which was pre-cooled to 4°C and cultured for 20 minutes at this temperature, followed by immunofluorescent staining.

### Morpholinos microinjection and RNA Interference

Oocytes were arrested at the GV stage in M2-containing 2.5 µM milrinone, 2 mM MK2 morpholino antisense oligos (GENE TOOLS, LLC, TCTGGCCCGGAGAGCCCGACAGCAT) microinjected into the cytoplasm to deplete MK2. The same amount of negative control morpholino (GENE TOOLS, LLC) was injected as control.

The GV-intact oocytes were microinjected in M2 medium containing 2.5 µM Milrinone with the negative control siRNA(Qiagen) and MK2 siRNAs (Ambion) (MK2-1 siRNA, GAACGAUGGGAGGAUGUCAtt) or (MK2-2 siRNA, ACAGAAUUCAUGAACCACCtt). The final concentration of the control or MK2 siRNA was 25 µM.

After microinjection, the oocytes were arrested at the GV stage for 24 h in M2-containing 2.5 µM milrinone to knock down MK2, and then transferred to Milrinone-free M16 medium to resume meiosis. Oocytes were incubated for different times and then collected for the subsequent experiments.

### Immunofluorescence and confocal microscopy

For staining of proteins, oocytes were fixed in 4% paraformaldehyde in PBS (pH 7.4) for at least 30 min at room temperature and processed for indirect immunofluorescence microscopy as described previously [70]. The immunostained cells were mounted on glass slides and examined with a Confocal Laser-Scanning Microscope (Zeiss LSM 510 META, Germany).

The following primary antibodies were used, respectively: rabbit anti-phospho-MAPKAPK-2 (Thr334; Cell Signaling; 1∶100), mouse anti-Plk1 antibody (Cell Signaling; 1∶50), mouse anti-γ-tubulin antibody (Sigma; 1∶200), mouse anti-α-tubulin-FITC antibody (Sigma; 1∶200), human anti-Crest antibody (Fitzgerald; 1∶50). Accordingly, the following secondary antibodies were used: FITC/TRITC-anti-mouse IgG (Zhong Shan Jin Qiao; 1∶100); FITC/TRITC-anti-rabbit IgG (Zhong Shan Jin Qiao; 1∶100); FITC-anti-human IgG (Zhong Shan Jin Qiao; 1∶100), Cy5-anti-human IgG (Jackson ImmunoResearch; 1∶200) or Cy5-anti-rabbit IgG (Jackson ImmunoResearch; 1∶200). Each experiment was repeated at least three times.

### Immunoblotting analysis

Mouse oocytes were collected in SDS sample buffer and heated for 5 min at 100°C. The proteins were separated by SDS-PAGE and then electrically transferred to polyvinylidene fluoride membranes. Following transfer, the membranes were blocked in TBST containing 5% skimmed milk for 2 h, followed by incubation overnight at 4°C with rabbit MK2 (1∶1000), rabbit cyclin B1 (BD Biosciences; 1∶500), and rabbit β-actin (1∶1000) antibodies, respectively. After washing three times in TBST, 10 min each, the membranes were incubated for 1 h at 37°C with 1∶1000 horseradish peroxidase-conjugated goat anti-rabbit IgG, respectively. Finally, the membranes were processed using the enhanced chemiluminescence detection system (Amersham, Piscataway, NJ).

### Preparation of chromosome spreads from mouse oocytes

For chromosome spreading, oocytes were kept for 20 minutes in 1% sodium citrate at room temperature and then fixed with fresh methanol: glacial acetic acid (3∶1). 10 mg/ml PI was used for chromosome staining [71]. Cells were examined with a Confocal Laser Scanning Microscope (Zeiss LSM 510 META, Germany).

### Statistical analysis

All percentages from at least three repeated experiments were expressed as means ±SEM, and the number of oocytes observed was labeled in parentheses as (n = ). Data were analyzed by paired-samples t-test. P<0.05 was considered statistically significant.

## Supporting Information

Figure S1 Subcellular localization of p-MK2 during mitosis. Hela cells grown on coverslips were immunostained with antibodies against rabbit p-MK2 (red), mouse α-tubulin (green), human Crest (purple) and labeled for DNA (blue). Each sample was counterstained with Hoechst 33258 to visualize DNA. Bar 5 µm. At prophase, p-MK2 was detected at chromosomes and microtubules. At prometaphase and metaphase, p-MK2 was detected at the chromosomes and spindles. At anaphase, p-MK2 disappeared from microtubules and was detected at chromosomes; at telophase, p-MK2 appeared as numerous dots associated with chromatin in the nucleus.
(9.83 MB TIF)

Figure S2 Depletion of MK2 by RNAi causes spindle assembly and chromosome alignment defects. (A) GV oocytes were microinjected with control siRNA and MK2-specific siRNA, respectively. After injection, oocytes were incubated in M16 medium containing 2.5 µM milrinone for 24 h, and then collected for western blotting (n = 150). (B) The rate of oocytes with first polar body in the control siRNA-injected group (n = 159) and MK2 siRNA2-injected group (n = 199). Data are presented as mean percentage (mean ± SEM) of at least three independent experiments. PB1, oocytes with the first polar body. Different superscripts denote statistical difference at a P<0.05 level of significance. (C) Spindle morphologies and chromosome alignment in control siRNA-injected oocytes and MK2 siRNA2-injected oocytes. After injection, oocytes were incubated in M16 medium containing 2.5 µM Milrinone for 24 h, and then transferred to Milrinone-free M16 for 16 h, followed by immunostaining with α-tubulin antibody (green) and with PI (red). In the control siRNA-injected group, normal bipolar spindles formed and chromosomes aligned correctly in the majority of oocytes (a). In the MK2 specific siRNA-injected group, various morphologically aberrant spindles and misaligned chromosomes are seen (b-d). Scale bar, 10 µm.
(9.75 MB TIF)

Figure S3 Re-culture of CMPD1 treated oocytes in fresh medium allows oocytes to resume meiosis progression. (A) The first polar body extrusion rates in different groups of oocytes cultured for various times. (−−):GV oocytes cultured in M16 medium with DMSO for 15 h; (++):GV oocytes cultured in M16 medium with CMPD1 for 15 h; (−+):GV oocytes cultured for 10 h, and then treated with CMPD1 for 5 h; (+−):GV oocytes cultured in M16 medium with CMPD1 for 10 h, and then cultured for 5 h after washing. Data are presented as mean percentage (mean ± SEM) of at least three independent experiments. (B) Spindle morphologies and chromosome alignment of oocytes. (++): GV oocytes were treated with CMPD1 for 15 h. (+−):GV oocytes cultured in M16 medium with CMPD1 for 10 h, and then in drug-free medium for 5 h. Oocytes were fixed and stained for p-MK2 (purple), α-tubulin (green) and DNA (red). Scale bar, 20 µm.
(9.88 MB TIF)

Figure S4 The speculative model chart of kinetochore microtubule attachment for control and MK2-deficient oocytes. In control oocytes at pro-MI stage, the two kinetochores of homologous chromosomes are captured by microtubules from opposite poles (amphitelic kinetochore orientation). At the MI stage, the homologous chromosomes are pulled under opposite microtubule tension. At AI, homologous chromosomes are separated by opposite pulling forces. At MII, the kinetochores of sister chromatids are captured by microtubules from opposite poles before separation. In MK2-deficient oocytes, at pro-MI stage, the kinetochores are not bound to microtubules (detached); one kinetochore is bound to microtubules, while the other kinetochore is not bound (monotelic kinetochore orientation); both kinetochores are bound to microtubules from the same pole (syntelic kinetochore orientation), or one kinetochore is bound to microtubules from both spindle poles (merotelic kinetochore orientation). Homologous chromosomes are not segregated under tension error, and the oocytes failed to enter anaphase.
(9.61 MB TIF)
