# Supplementary figures and images for: MAPK-Activated Protein Kinase 2 Is Required for Mouse Meiotic Spindle Assembly and Kinetochore-Microtubule Attachment
Source: PLoS One. 2010 Jun 28;5(6):e11247. doi: 10.1371/journal.pone.0011247 (PMC2893158; doi:10.1371/journal.pone.0011247)

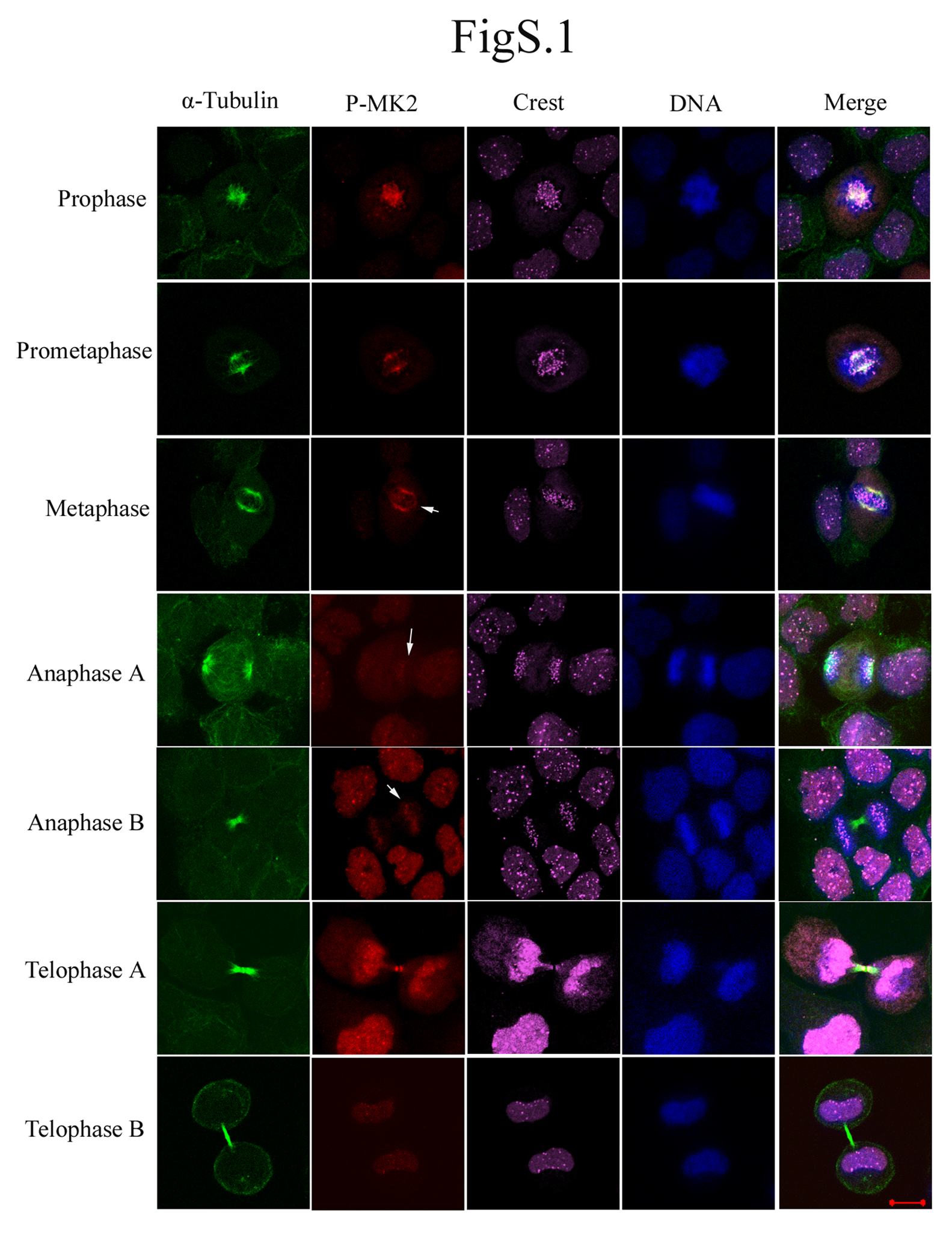

Supplement: Figure S1 — Subcellular localization of p-MK2 during mitosis. Hela cells grown on coverslips were immunostained with antibodies against rabbit p-MK2 (red), mouse α-tubulin (green), human Crest (purple) and labeled for DNA (blue). Each sample was counterstained with Hoechst 33258 to visualize DNA. Bar 5 µm. At prophase, p-MK2 was detected at chromosomes and microtubules. At prometaphase and metaphase, p-MK2 was detected at the chromosomes and spindles. At anaphase, p-MK2 disappeared from microtubules and was detected at chromosomes; at telophase, p-MK2 appeared as numerous dots associated with chromatin in the nucleus. (9.83 MB TIF) [file pone.0011247.s001.tif]

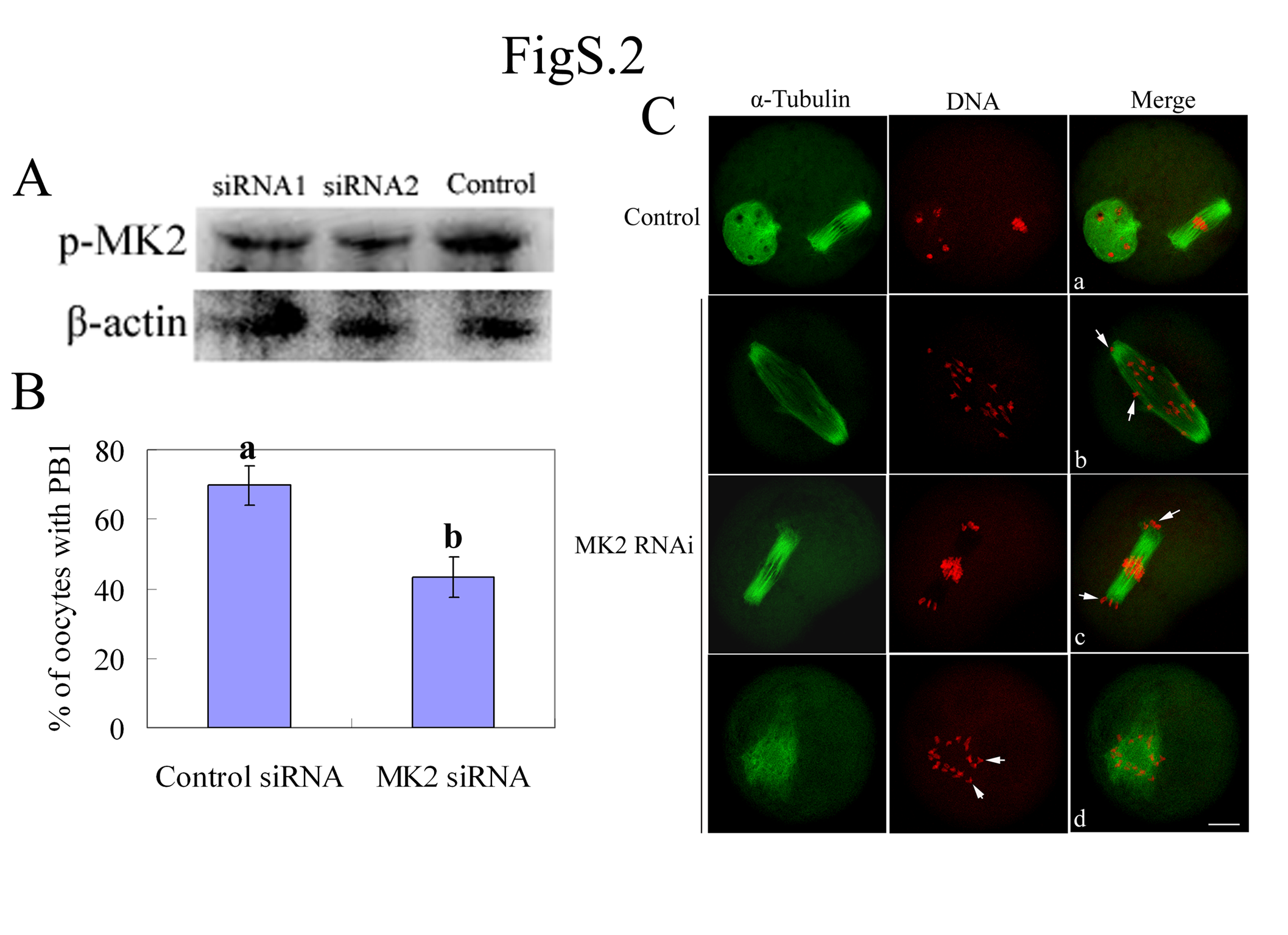

Supplement: Figure S2 — Depletion of MK2 by RNAi causes spindle assembly and chromosome alignment defects. (A) GV oocytes were microinjected with control siRNA and MK2-specific siRNA, respectively. After injection, oocytes were incubated in M16 medium containing 2.5 µM milrinone for 24 h, and then collected for western blotting (n = 150). (B) The rate of oocytes with first polar body in the control siRNA-injected group (n = 159) and MK2 siRNA2-injected group (n = 199). Data are presented as mean percentage (mean ± SEM) of at least three independent experiments. PB1, oocytes with the first polar body. Different superscripts denote statistical difference at a P<0.05 level of significance. (C) Spindle morphologies and chromosome alignment in control siRNA-injected oocytes and MK2 siRNA2-injected oocytes. After injection, oocytes were incubated in M16 medium containing 2.5 µM Milrinone for 24 h, and then transferred to Milrinone-free M16 for 16 h, followed by immunostaining with α-tubulin antibody (green) and with PI (red). In the control siRNA-injected group, normal bipolar spindles formed and chromosomes aligned correctly in the majority of oocytes (a). In the MK2 specific siRNA-injected group, various morphologically aberrant spindles and misaligned chromosomes are seen (b-d). Scale bar, 10 µm. (9.75 MB TIF) [file pone.0011247.s002.tif]

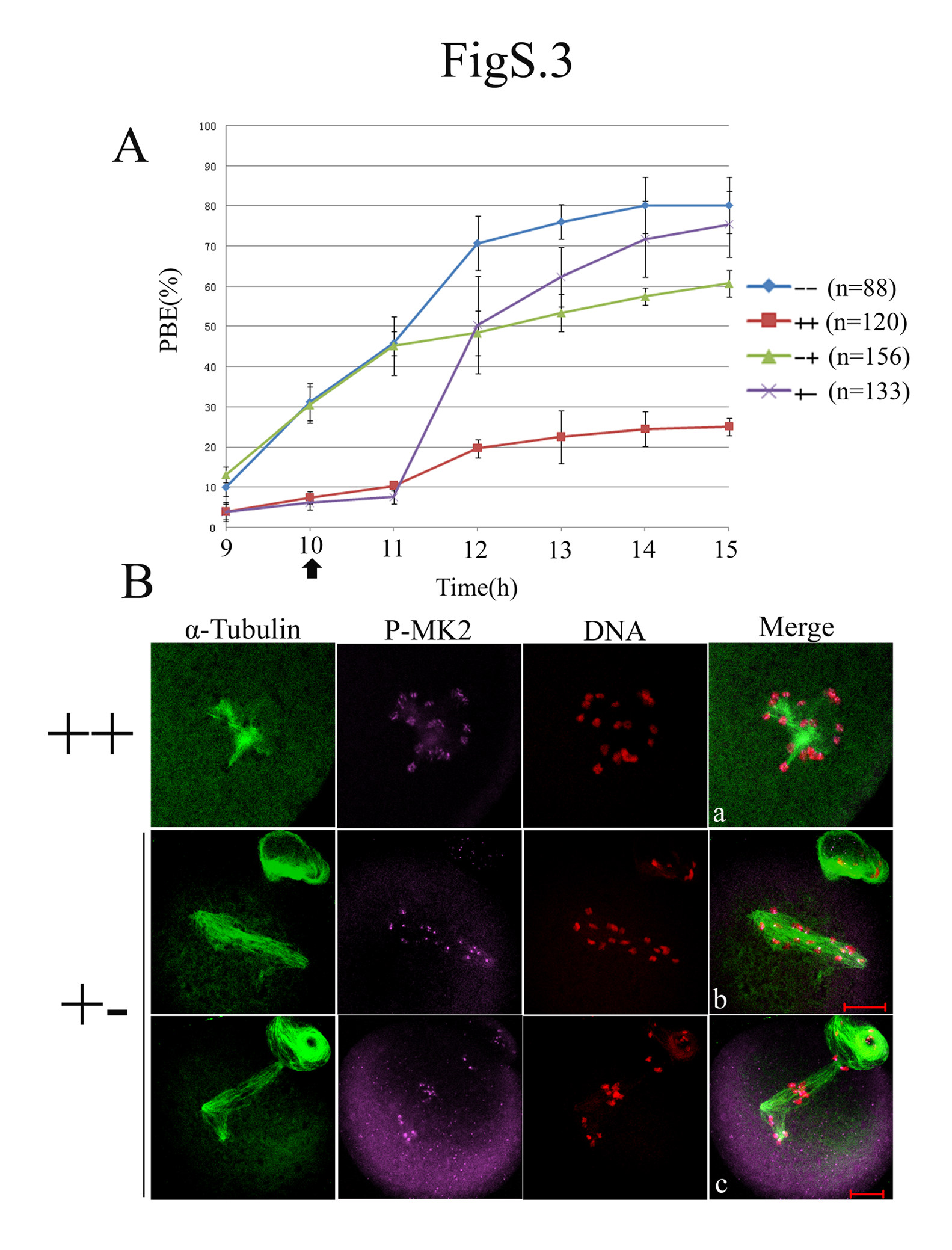

Supplement: Figure S3 — Re-culture of CMPD1 treated oocytes in fresh medium allows oocytes to resume meiosis progression. (A) The first polar body extrusion rates in different groups of oocytes cultured for various times. (−−):GV oocytes cultured in M16 medium with DMSO for 15 h; (++):GV oocytes cultured in M16 medium with CMPD1 for 15 h; (−+):GV oocytes cultured for 10 h, and then treated with CMPD1 for 5 h; (+−):GV oocytes cultured in M16 medium with CMPD1 for 10 h, and then cultured for 5 h after washing. Data are presented as mean percentage (mean ± SEM) of at least three independent experiments. (B) Spindle morphologies and chromosome alignment of oocytes. (++): GV oocytes were treated with CMPD1 for 15 h. (+−):GV oocytes cultured in M16 medium with CMPD1 for 10 h, and then in drug-free medium for 5 h. Oocytes were fixed and stained for p-MK2 (purple), α-tubulin (green) and DNA (red). Scale bar, 20 µm. (9.88 MB TIF) [file pone.0011247.s003.tif]

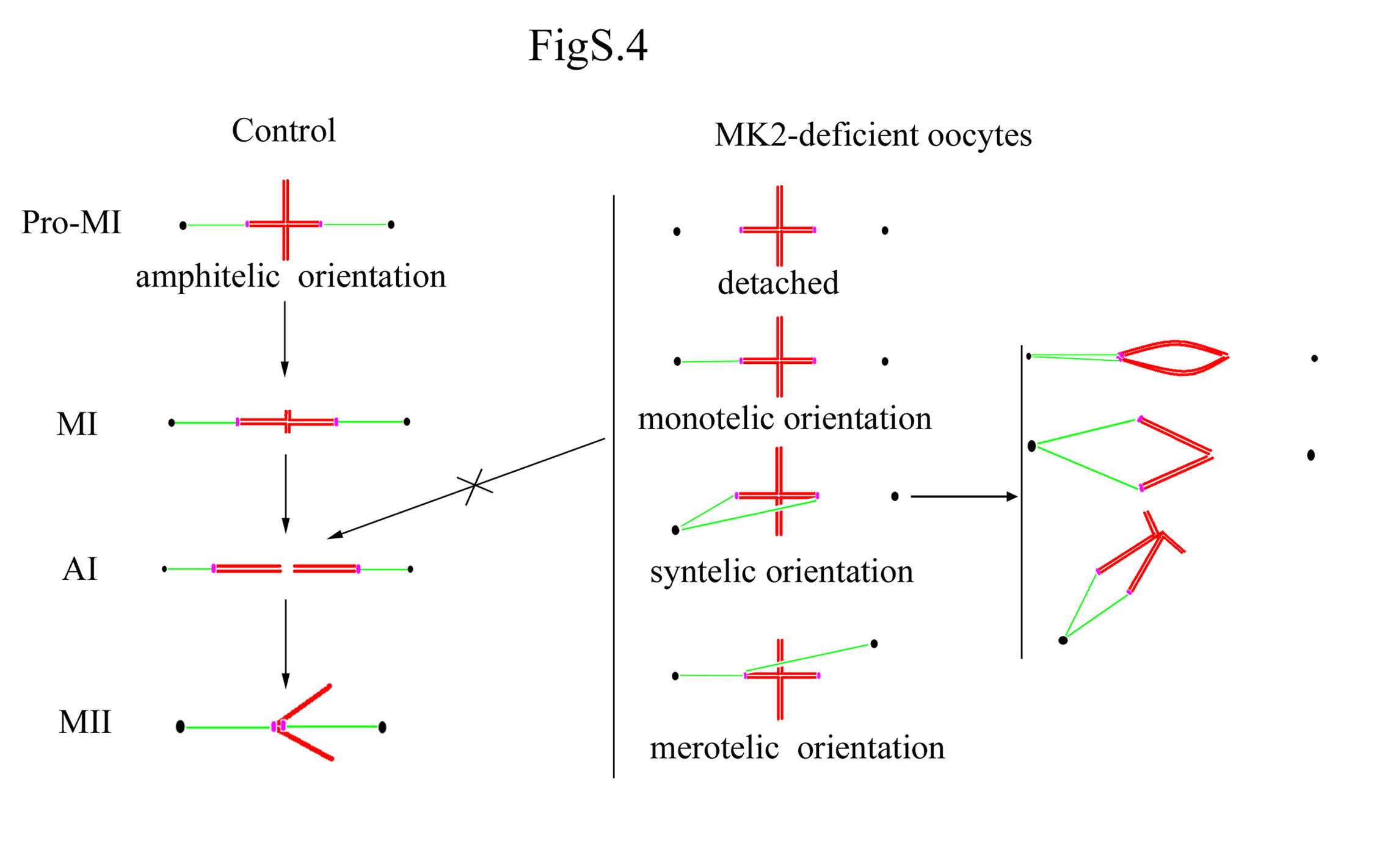

Supplement: Figure S4 — The speculative model chart of kinetochore microtubule attachment for control and MK2-deficient oocytes. In control oocytes at pro-MI stage, the two kinetochores of homologous chromosomes are captured by microtubules from opposite poles (amphitelic kinetochore orientation). At the MI stage, the homologous chromosomes are pulled under opposite microtubule tension. At AI, homologous chromosomes are separated by opposite pulling forces. At MII, the kinetochores of sister chromatids are captured by microtubules from opposite poles before separation. In MK2-deficient oocytes, at pro-MI stage, the kinetochores are not bound to microtubules (detached); one kinetochore is bound to microtubules, while the other kinetochore is not bound (monotelic kinetochore orientation); both kinetochores are bound to microtubules from the same pole (syntelic kinetochore orientation), or one kinetochore is bound to microtubules from both spindle poles (merotelic kinetochore orientation). Homologous chromosomes are not segregated under tension error, and the oocytes failed to enter anaphase. (9.61 MB TIF) [file pone.0011247.s004.tif]
